# Effects of Different Recovery Positions on the Postpercutaneous Liver Biopsy Complications: A Metaanalysis

**DOI:** 10.3389/fsurg.2021.707945

**Published:** 2021-10-29

**Authors:** Chengli Zhong, Jiandi Jin, Xiaoyan Wang, Yandi Huang, Dong Yan, Wei Wang

**Affiliations:** ^1^State Key Laboratory for Diagnosis and Treatment of Infectious Diseases, National Clinical Research Center for Infectious Diseases, National Medical Center for Infectious Diseases, Collaborative Innovation Center for Diagnosis and Treatment of Infectious Diseases, College of Medicine, The First Affiliated Hospital, Zhejiang University, Hangzhou, China; ^2^Department of Laboratory Medicine, The First Affiliated Hospital, Zhejiang University School of Medicine, Hangzhou, China; ^3^The First Affiliated Hospital, Zhejiang University School of Medicine, Hangzhou, China

**Keywords:** abdominal bleeding, complications, liver biopsy, meta-analysis, percutaneous, postoperative position

## Abstract

**Objective:** We aim to evaluate the effects of different recovery positions on the adverse events and the patient acceptability in those who underwent percutaneous liver biopsy (PLB).

**Methods:** A literature search was conducted in the Cochrane Library, Embase, Scopus, PubMed, CNKI, Sinomed, and Wanfang databases. The time for the article extraction was until July 2020. The articles were screened by two independent researchers, together with the bias risk evaluation and data extraction. The RevMan 5.4 software was utilized for the metaanalysis.

**Results:** Finally, two articles involving 180 subjects were eligible for this study. Metaanalysis showed that at T0, the alternation between right-side and combined position (CRP) would induce an elevation of post-PLB pain compared with the dorsal/supine position (SRP) [WMD = −2.00, 95% CI (−3.54, −0.47), *p* = 0.01]. There were no statistical differences in the postoperative pain among the CRP, SRP, and right-side position (RRP). The patient acceptability of SRP and RRP was higher than that of the CRP. Finally, two eligible studies were included, which showed no incidence of pneumothorax and abdominal bleeding.

**Conclusions:** CRP would induce post-PLB pain at T0. SRP was the most acceptable position for the cases that underwent PLB. There were no statistical differences in the incidence of pneumothorax and abdominal bleeding.

**Systematic Review Registration:** https://www.crd.york.ac.uk/PROSPERO, identifier: CRD42020196633.

## Introduction

**Figure 1:**
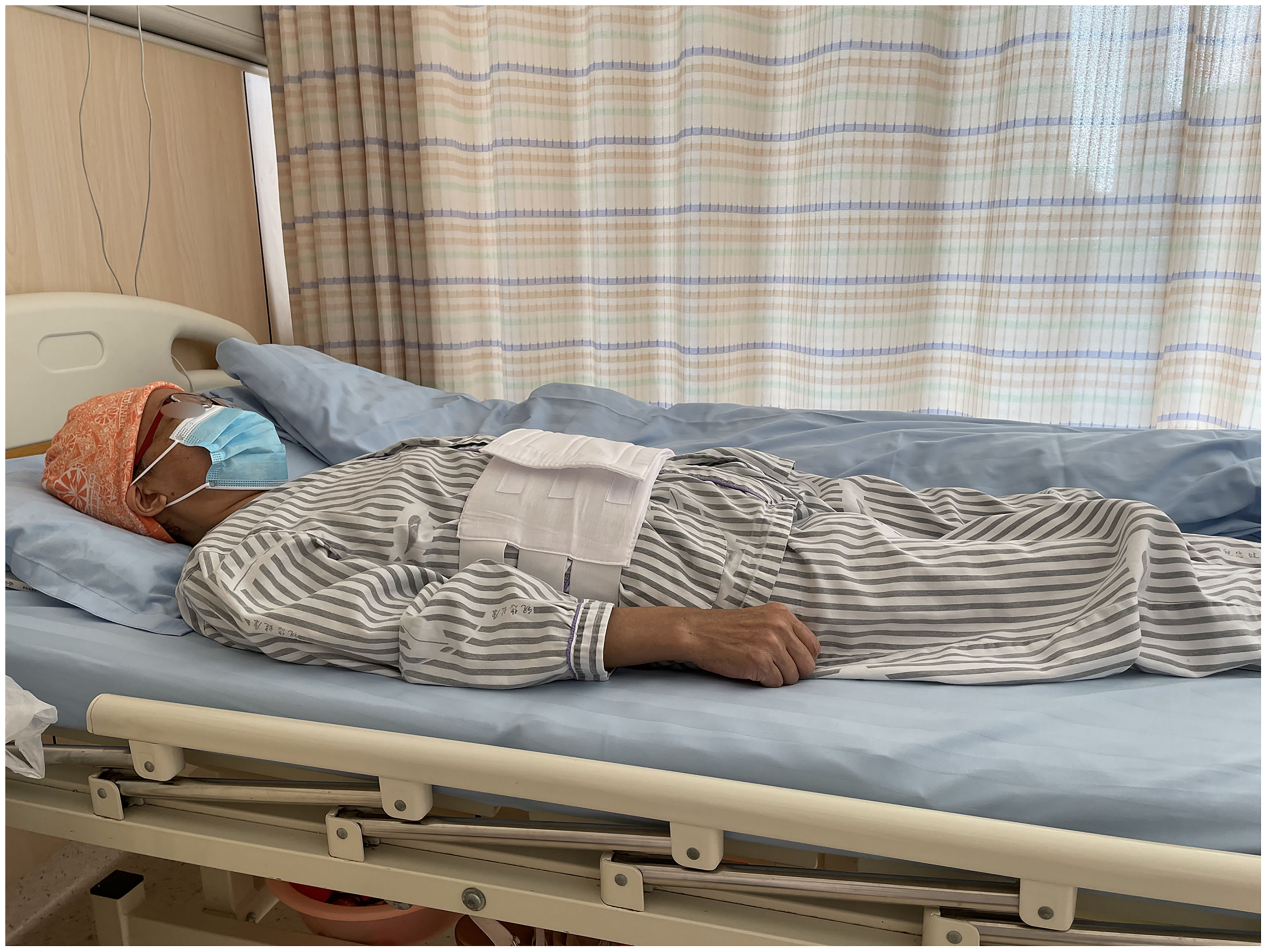
Supine position (SRP).

**Figure 2:**
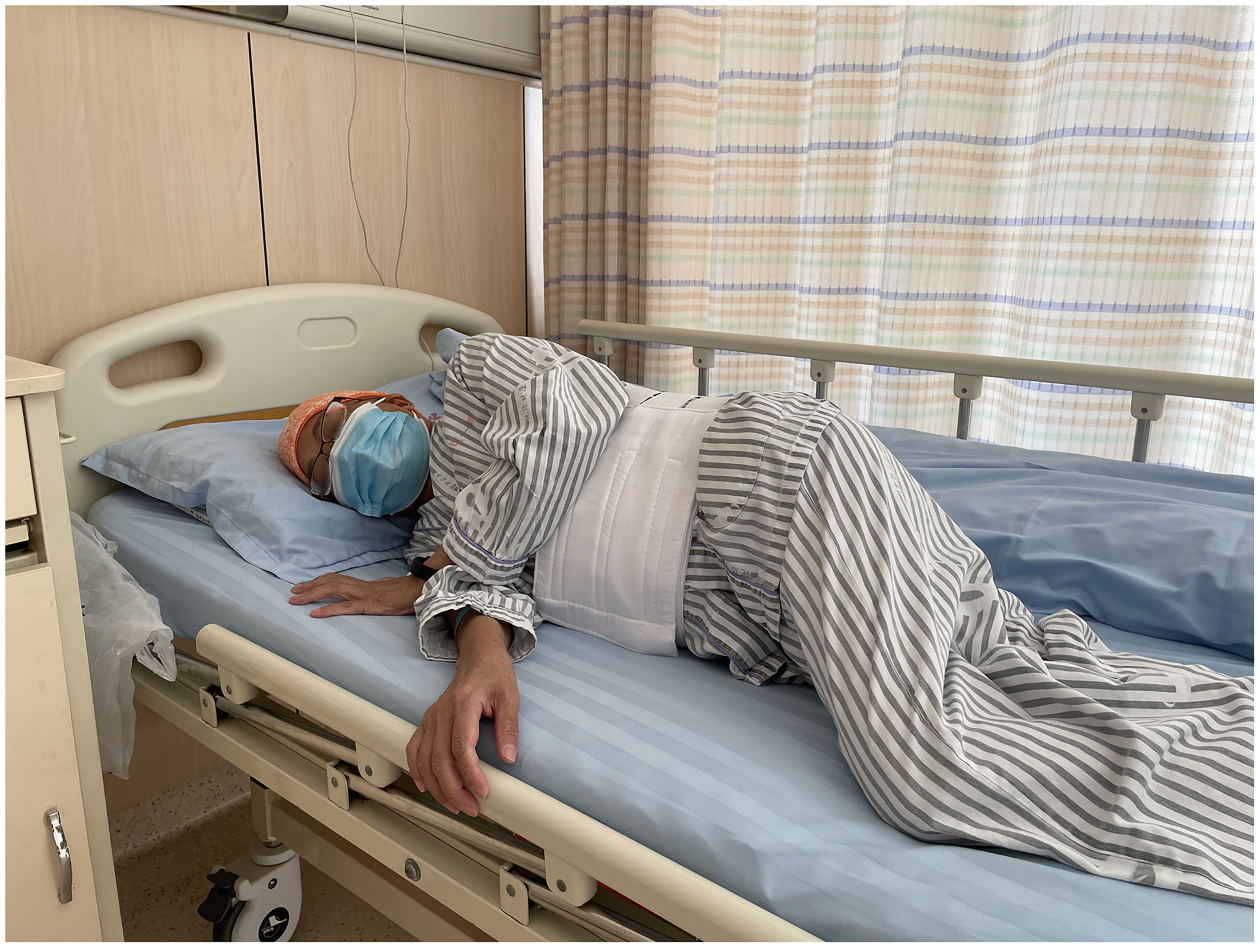
Right side position (RRP).

**Figure 3:**
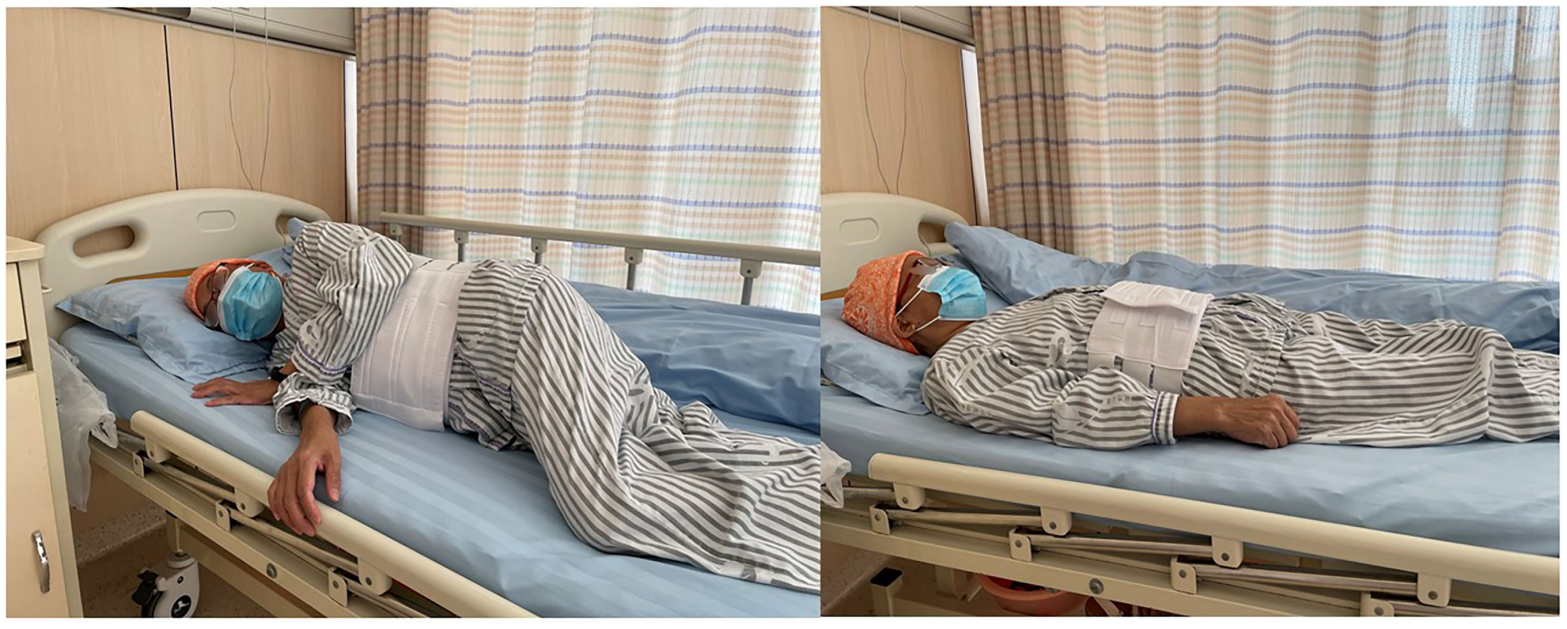
The alternation between right-side and combined position (CRP): right lateral decubitus for 2 h, followed by supine position for 1 h.

Percutaneous liver biopsy (PLB), serves as a tool for the evaluation of liver injury and/or fibrosis and has been commonly utilized for the diagnosis, treatment, and outcome prediction of hepatic diseases (1, 2). Nowadays, it has been considered a conventional surgery with high safety (3, 4), with total mortality of 0.2% (5, 6), as well as a hemorrhagic tendency of 0.01–0.5% (5, 7, 8). However, there is still no consensus on the recovery position after surgery, which may lead to confusion among surgeons and physicians (9). Currently, the post-PLB recovery position includes dorsal/supine position (SRP) (Figure 1), right-side position (RRP) (Figure 2), and the alternation between right-side and combined position (CRP) (10–12) (Figure 3). To date, the patients were suggested to lie on their right (RRP) for 2 h, followed by SRP for 1 h. In the 2020 BSG/RCR/RCP guidelines, those who underwent PLB were recommended to lie on RRP for 3 h (13). This information contributed to the caring and management of patients but induced confusion due to a lack of a standard for the post-PLB recovery position (9, 12, 14). In this study, we aimed to investigate the effects of different recovery positions on adverse events and patient acceptability, to provide an evidence-based standard for the recovery position after PLB.

## Materials and Methods

### Study Eligibility

Articles which met the following criteria were eligible for this study: (i) those who received PLB with an age of ≥18-year-old received PLB; (ii) randomized controlled trials (RCTs), case-control studies, or cohort studies; (iii) articles published in Chinese and/or English; and (iv) articles that reported the pain and patient compliance. The articles with a duplicate publication, full-article not available, or challenges in the data extraction were excluded. This study was registered on the PROSPERO website (registration no.: CRD42020196633).

### Literature Search

A literature search was conducted in the Cochrane Library, Embase, Scopus, PubMed, CNKI, Sinomed, and Wanfang databases. The keywords used in the search were as follows: “position/decub/posture,” “lateral/ side /prone,” “biopsy/aspiration/puncture,” and “liver/livers/he par/hepatic,”

### Data Extraction and Quality Control

Two authors independently reviewed the articles based on the inclusion and exclusion criteria. The extracted data included: (i) author, year of publication, and country; (ii) age and sample size; (iii) time and frequency for the interference; (iv) and outcome indices. The evaluation was conducted based on the standards proposed in the Cochrane manual (5.1.0 version). The evaluated items involved seven questions, which were categorized into “low risk bias,” “high risk bias,” and “unknown” for each standard, respectively. Those who met the seven standards completely showed a small possibility of bias and were listed as grade A for the quality control. Those who met 1–6 standards present a possibility of moderate bias and were listed as grade B. Those who met none of the standards were listed as grade C and presented a high possibility of bias. In this study, we only included articles evaluated as grades A and B. In cases of any disputes between the two authors, deep communication was held after consulting the third staff experienced in this field (Table 1).

**Table 1:** Basic information of the eligible studies.

**References**	**Year of publication**	**Nation**	**Sample size**	**RRP**	**SRP**	**CRP**	**Interference time, h**
Hyun et al. (11)	2005	US	90	27	32	31	4
Costa et al. (10)	2019	Portugal	90	27	33	30	4

### Statistical Analysis

The RevMan 5.4 software was utilized for the metaanalysis. The weighted mean difference (WMD) or the standard mean difference (SMD) was used as the effector index. The 95% confidence interval (CI) indicated the effect size. The Chi square test was adopted to testify the heterogeneity. Homogeneity was considered in the presence of *p* > 0.1 and *I*^2^ < 50%, and then the fixed effect model was selected for the analysis. In cases of *p* < 0.1 and *I*^2^ ≥ 50%, the source of the heterogeneity was analyzed. If there was no significant clinical heterogeneity, the random effect model was adopted for the analysis.

## Results

### Eligible Studies

**Figure 4:**
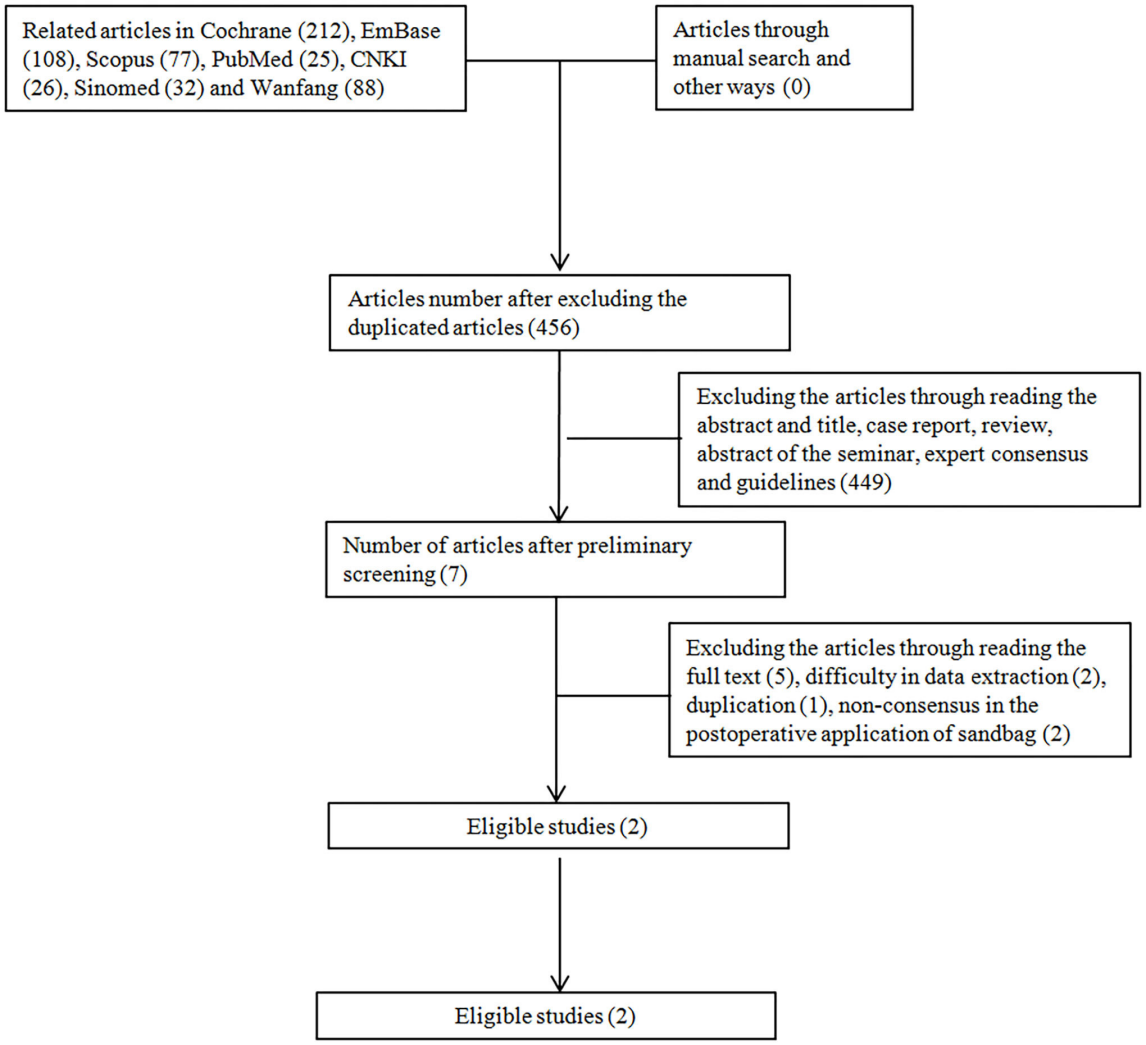
Flowchart of the study.

In this study, a total of 568 articles were found after the literature search, which yielded 456 articles after the elimination of duplicated publications. Then, we excluded seven articles after reading the title and the abstract, which generated 449 articles. Five articles were excluded due to incompliance in the research type, two due to incompliance of subjects, and one due to duplicated publication. Finally, two English articles were included involving 180 subjects (Figure 4).

### Basic Features and the Bias Evaluation for the Eligible Studies

Finally, two English articles comprising 180 cases were included. The quality of the articles was evaluated using the Cochrane manual (5.1.0 software). The two articles were classified as grade A (Table 2).

**Table 2:** Methodology evaluation for the eligible studies.

**References**	**Random sequence**	**Occult distribution**	**Blinded study**		**Data integrity**	**Results for the selective report**	**Other bias**	**Quality grade**
			**To the subjects or patients**	**To these underwent data evaluation**				
Hyun et al. (11)	High	High	High	High	High	High	Low	A
Costa et al. (10)	High	High	High	High	High	High	Low	A

### Metaanalysis

#### The Effects of Recovery Position on the Pain at Time 0 (T0)

**Figure 5:**
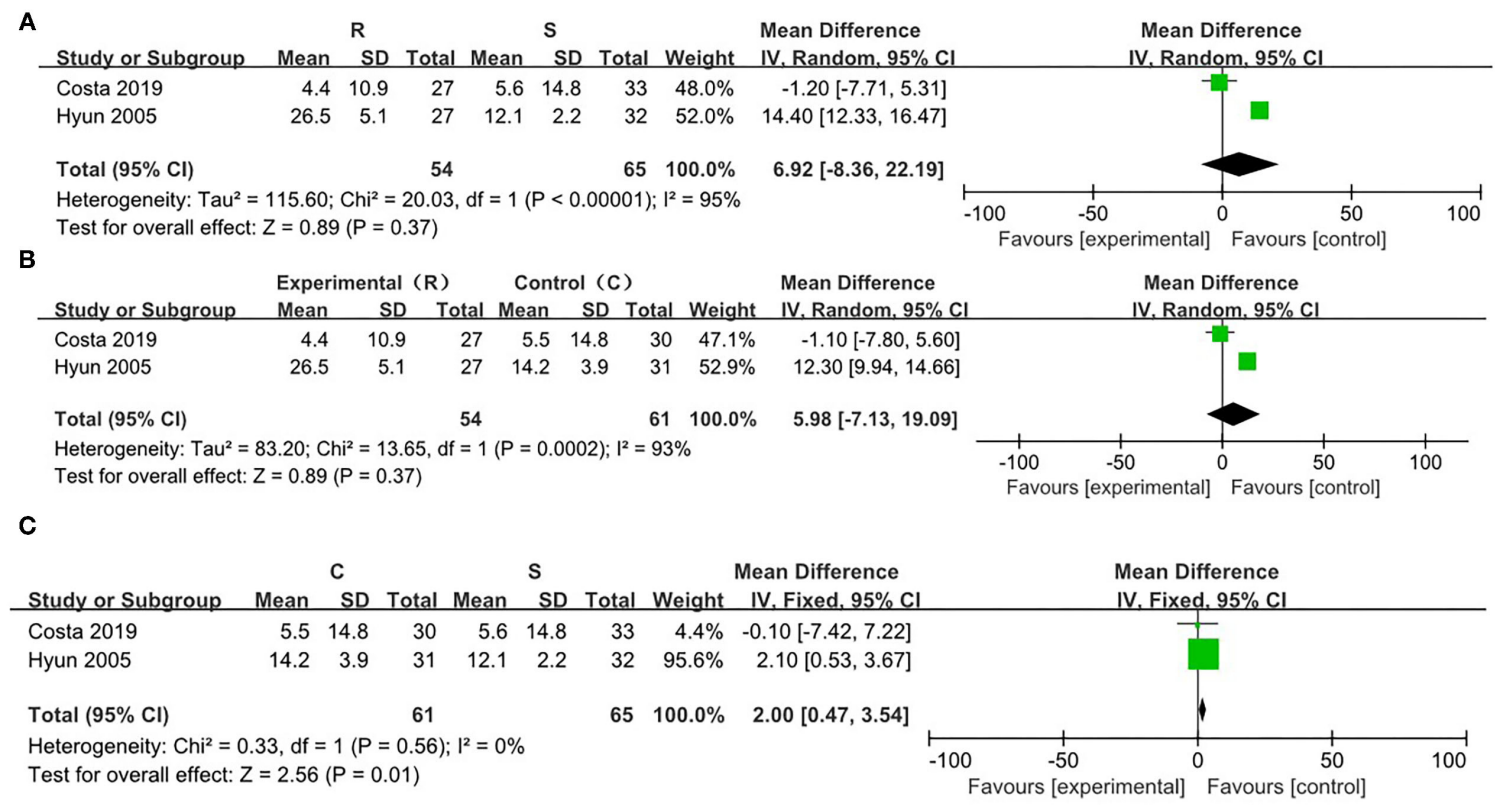
Risk evaluation for the bias of the literatures at T0. **(A)** CRP vs. SRP; **(B)** RRP vs SRP; **(C)** RRP vs. CRP.

Compared with the SRP, there was significant heterogeneity in the RRP (χ^2^ = 55.71, *p* < 0.00001, *I*^2^ = 98%). On this basis, a random effect model was utilized. There were no significant differences in the attenuation of the postoperative pain between the cases with the SRP and the RRP [WMD = −1.80, 95% CI (−1.94, 5.55), *p* = 0.34] (Figure 5A).

In comparison between the RRP and CRP, no significant heterogeneity was found (χ^2^ = 13.55, *p* = 0.0002, *I*^2^ = 93%). On this basis, the random effect model was adopted. There were no statistical differences in the attenuation of the postoperative pain between the two positions [WMD = −5.99, 95% CI (−7.12, 19.10), *p* = 0.37] (Figure 5B).

On comparing SRP and CRP, no significant heterogeneity was found (χ^2^ = 0.33, *p* = 0.56, *I*^2^ = 0%). On this basis, the fixed effect model was adopted. There were statistical differences in the attenuation of post-PLB pain between the two positions [WMD = −2.00, 95% CI (−3.54, −0.47), *p* = 0.01] (Figure 5C).

#### The Effects of Recovery Position on the Pain at T2

**Figure 6:**
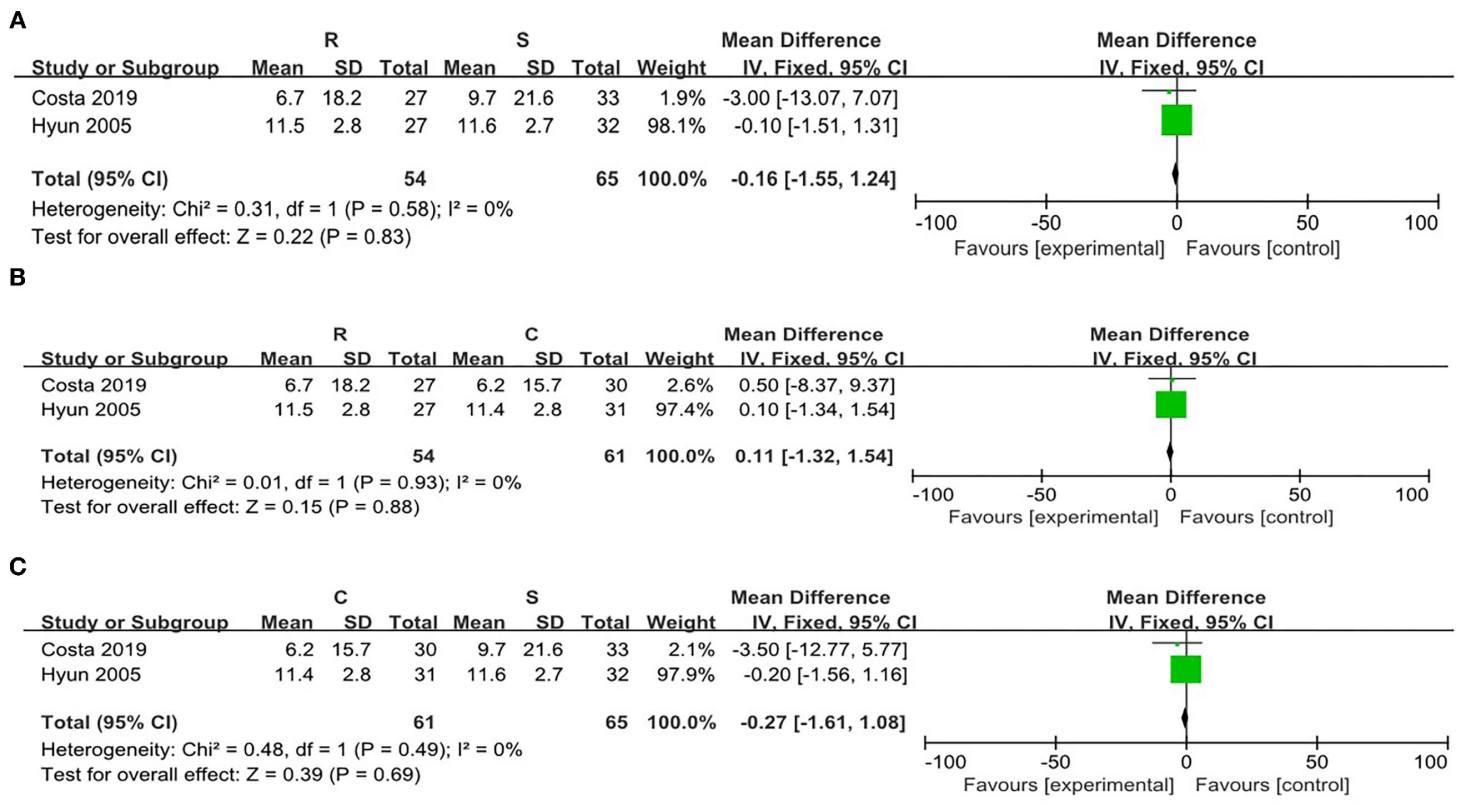
Risk evaluation for the bias of the literatures at T2. **(A)** CRP vs. SRP; **(B)** RRP vs SRP; **(C)** RRP vs. CRP.

There was no significant heterogeneity between the SRP and the RRP (χ^2^ = 3.17, *p* = 0.08, *I*^2^ = 68%). Thus, the fixed effect model was adopted. The data showed that there were no significant differences in the attenuation of the post-PLB between the two positions [WMD = −0.32, 95% CI (−6.7, 6.06), *p* = 0.92] (Figure 6A).

When comparing the RRP and the CRP, no significant heterogeneity was found (χ^2^ = 8.18, *p* = 0.004, *I*^2^ = 88%). Thus, the random effect model was adopted. There were no statistical differences in the post-PLB pain between the two positions [WMD = −6.20, 95% CI (−14.10, 1.71)] (Figure 6B).

Compared with the SRP, there was significant heterogeneity in the CRP (χ^2^ = 12.48, *p* = 0.004, *I*^2^ = 92%). Thus, the random effect model was adopted. There were no statistical differences in the attenuation of post-PLB pain between the two positions [WMD = −5.08, 95% CI (−19.64, 9.49), *p* = 0.49] (Figure 6C).

#### The Effects of Recovery Position on the Pain at T4

**Figure 7:**
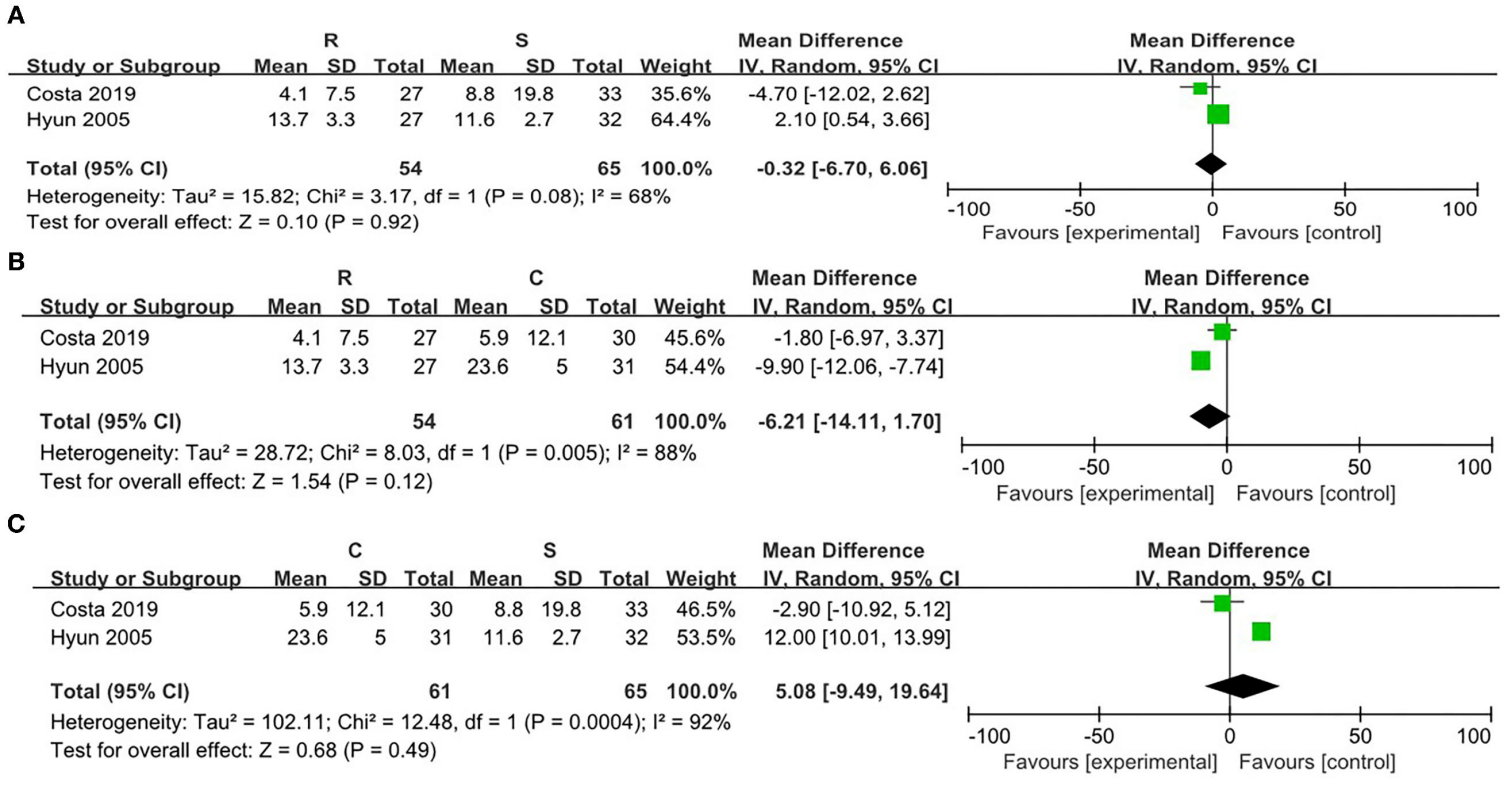
Risk evaluation for the bias of the literatures at T4. **(A)** CRP vs. SRP; **(B)** RRP vs SRP; **(C)** RRP vs. CRP.

There was no significant heterogeneity between the SRP and the RRP (χ^2^ = 0.31, *p* = 0.58, *I*^2^ = 0%). On this basis, the fixed effect model was adopted. There were no significant differences in the attenuation of the post-PLB between the two positions [WMD = −0.16, 95% CI (−1.55, 1.24), *p* = 0.83] (Figure 7A).

When comparing the RRP and the CRP, no significant heterogeneity was found (χ^2^ = 0.01, *p* = 0.93, *I*^2^ = 0%). Then fixed effect model was adopted. There were no statistical differences in the post-PLB pain between the two positions [WMD = 0.11, 95% CI (−1.32, 1.54), *p* = 0.88] (Figure 7B).

On comparison between the SRP and the CRP, no significant heterogeneity was found (χ^2^ = 0.48, *p* = 0.49, *I*^2^ = 0%). Therefore, the fixed effect model was utilized. There were no statistical differences in the post-PLB pain between the SRP and the CRP [WMD = −0.27, 95% CI (−1.08, 1.61), *p* = 0.69] (Figure 7C).

#### PLB Position Compliance in the Cases

Both RCTs included in this metaanalysis focused on the compliance of the PLB position. Costa et al. indicated that the RRP group considered the procedure less acceptable than the SRP group (*p* = 0.001) or the combined group (*p* = 0.002). There was no difference between the SRP and the CRP groups (*p* = 0.77). Hyun et al. evaluated the overall acceptability of the biopsy experience based on the visual simulation, which indicated that the acceptability in the combined group was significantly lower than that of the supine position (89.2 ± 2.6 vs. 94.5 ± 1.4, *p* = 0.047) and the RRR group (89.2 ± 2.6 vs. 94.7 ± 1.4, *p* = 0.046). As the data extracted from Costa et al. were numeration data and those from Hyun et al. were measurement data, we could not conduct the metaanalysis. Nevertheless, we could confirm that the supine position was more commonly accepted by the patients.

#### Effects of Post-PLB Recovery Position on Other Complications

In the two trials, there were no severe adverse events such as pneumothorax and abdominal hemorrhage. Costa et al. reported that 38 cases (42.2%) showed at least one complication, mainly pain. Thirty-six cases reported pain in the recovery phage (1–7 score). Two cases showed pain in the vasovagal reaction. Twenty-five showed spontaneous relief in the pain. Ten (11.1%) received an intravenous injection of paracetamol, and two cases received an intravenous injection of pethidine for the pain analgesia. Three cases showed vomiting and nausea.

## Discussion

Patients who underwent PLB are advised to receive a combined position by UpToDate including an RRR position for 2 h followed by SRP for 1 h; however, an RRR position for 3 h is proposed by the 2020 BSG/RCR/RCP guideline 13. Up until now, there is still no consensus on the effects of the recovery position on the post-PLB complications and patient acceptability. On this basis, there are variations in the post-PLB management in different hospitals. This induced confusion for the procedure selection in clinical settings (15). In this study, the two articles included in the metaanalysis reported the inclusion and exclusion criteria, together with the diagnostic method and post-PLB recovery position. Therefore, the results of the metaanalysis showed high reliability.

Pain is the most common complication of PLB (16, 17), with about 25% of the cases presenting pain in the right upper abdomen or right shoulder (1, 15, 18). Particularly, those (1–5%) with moderate or severe pain should be admitted to a hospital for further treatment (19). The combined position was proposed by UpToDate as it was speculated to exert compressive effects; however, there are no evidence-based proofs for it. For those who underwent PLB, a supine position for at least 3 h was proposed by the British Society of Gastroenterology; however, there is still no evidence for it (13). In this study, we enrolled two RCTs, and then the metaanalysis was conducted, which showed that the post-PLB pain score in the supine position was significantly lower than that of the combined position at T0. This may be related to the local stimulation-induced pain sensation by RRP or during the body position changes (10, 11). There were no statistical differences in the post-PLB pain score among the patients in the supine position, RRP, and CRP at T2 and T4, respectively. Thus, the CRP was not the ideal position for the recovery, which may be related to the post-PLB pain induced by the body position (11). For the supine position, it involved less changes in the body position and was less affected by the interference factors. Therefore, fewer cases showed pain. The pathogenesis of pain was associated with the fact that the hemorrhage induced by a puncture would stimulate the abdomen and/or gallbladder. In a previous study, laparoscopic hemostasis contributed to the decline of pain (20). Besides, the ultrasonic-guided PLB induced a decline of 30% in pain among those who received PLB using an automatic cutting needle (21, 22). This may be related to the fact that the ultrasonic-guided puncture would avoid any damages to the vessels, which reduced the possibility of hemorrhage. In addition, the puncture may not be performed after a try. The application of a nitrous oxide system would reduce puncture-induced pain (14). In cases of administration of fentanyl and dormicum, the ultrasonic-guided puncture would attenuate the pain and discomforts (23).

To evaluate the treatment efficiency, PLB should be given twice or more. Poor acceptability is a response to the refusal of the second PLB. Specifically, about 41% of the cases showed poor acceptability for the PLB, and about 6–9% of them refused to receive PLB again (24, 25). In this study, the two RCTs investigated the acceptability of the patients to the different body positions for PLB after discharge. Costa et al. indicated that the right-side position group considered the procedure less acceptable than the DRP group or the combined group. In addition, there was no difference between the DRP and the CRP groups. Hyun et al. indicated that the combined group was significantly lower than that in the supine position and the RRP. As the data extracted from Costa et al. were numeration data and these from Hyun et al. were measurement data, we could not conduct the metaanalysis. Nevertheless, we could confirm that the supine position was more commonly accepted by the patients. In a large clinical trial, the incidence of complications induced by multiple punctures was significantly higher than that induced by a single puncture. Venous channel and the handling of the procedures were risk factors for the discomforts among these patients (25). Multiple punctures would reduce the willingness of the patients to undergo the PLB (25).

In a previous study, Chris et al. reported no other severe complications except pain (11). In contrast, Rita et al. reported the incidence of vasovagal reaction in two cases (2.2%) (10), which was in line with the previous study (17, 25). As the two RCTs enrolled in this study involved inadequate cases, we did not observe any complications (e.g., hemorrhage). As there were less severe post-PLB complications, the two RCTs did not focus on the complication analysis. According to the previous description, the incidence of hemorrhage after PLB was less than merely 0.3%, which usually took place within 1 h after the puncture. In contrast, the incidence of subclinical hemorrhage after ultrasonography was up to 23% (26).

Indeed, there are some limitations to our study. There are few studies on the recovery position after PLB, and the number of articles in this study is limited, due to which the risk factors for the post-PLB pain and complications could not be completely identified. Nowadays, RRR and combined position are commonly used, while in mainland China, most of the studies utilize the supine position. Meanwhile, as the sample size is too small, we could not evaluate the adverse events such as hemorrhage, pneumothorax, or even death. In the future, more studies involving a large sample size and high-quality prospective analysis are required to further illustrate the safety of the recovery position after PLB.

This metaanalysis indicated that PLB was a safe procedure with good acceptability by the patients. The major adverse event included the mild pain that was spontaneously relieved. No other major complications were observed. post-PLB combined position would increase the possibility of pain. The supine position and the RRR were the most acceptable recovery positions for the patients. For the combined position, there must be body position changes within 2 h, which may lead to discomforts and poor compliance. Therefore, the supine position was the most acceptable recovery position with good comfort.

## Data Availability Statement

The original contributions presented in the study are included in the article/supplementary material, further inquiries can be directed to the corresponding author/s.

## Author Contributions

The manuscript has been written by CZ and revised by WW and DY. Credit goes to XW and YH for the collection of the necessary data. Data analysis was performed by JJ. All authors have read and approved the final manuscript.

## Funding

This work was supported by National Science and Technology Major Project (2018ZX10101-001).

## Conflict of Interest

The authors declare that the research was conducted in the absence of any commercial or financial relationships that could be construed as a potential conflict of interest.

## Publisher's Note

All claims expressed in this article are solely those of the authors and do not necessarily represent those of their affiliated organizations, or those of the publisher, the editors and the reviewers. Any product that may be evaluated in this article, or claim that may be made by its manufacturer, is not guaranteed or endorsed by the publisher.

## Abbreviations

PLB: percutaneous liver biopsy
CRP: combined position
SRP: supine position
RRP: right side position
WMD: weighted mean difference
SMD: standard mean difference
CI: confidence interval.

## References

[1] Rockey DC Caldwell SH Goodman ZD Nelson RC Smith AD. Liver biopsy. Hepatology. (2009) 49:1017–44. 10.1002/hep.22742 19243014

[2] EASL-ALEH clinical practice guidelines: non-invasive tests for evaluation of liver disease severity and prognosis. J Hepatol. (2015) 63:237–64. Epub 2015/04/26. PubMed PMID: 25911335. 10.1016/j.jhep.2015.04.006 25911335

[3] Pinelo E Presa J. Outpatient percutaneous liver biopsy: still a good option. Eur J Intern Med. (2009) 20:487–9. 10.1016/j.ejim.2009.02.002 19712850

[4] Janes CH Lindor KD. Outcome of patients hospitalized for complications after outpatient liver biopsy. Ann Intern Med. (1993) 118:96–8. 10.7326/0003-4819-118-2-199301150-00003 8416324

[5] West J Card TR. Reduced mortality rates following elective percutaneous liver biopsies. Gastroenterology. (2010) 139:1230–7. 10.1053/j.gastro.2010.06.015 20547160

[6] Sparchez Z. Complications after percutaneous liver biopsy in diffuse hepatopathies. Rom J Gastroenterol. (2005) 14:379–84. 16400355

[7] Boyum JH Atwell TD Schmit GD Poterucha JJ Schleck CD Harmsen WS . Incidence and Risk Factors for Adverse Events Related to Image-Guided Liver Biopsy. Mayo Clin Proc. (2016) 91:329–35. 10.1016/j.mayocp.2015.11.015 26837481

[8] Huang JF Hsieh MY Dai CY Hou NJ Lee LP Lin ZY . The incidence and risks of liver biopsy in non-cirrhotic patients: an evaluation of 3806 biopsies. Gut. (2007) 56:736–7. 10.1136/gut.2006.115410 17440193 PMC1942123

[9] Wilkinson MM. Your role in needle biopsy of the liver. RN. (1990) 53:62–6. 2385790

[10] Costa RS Cardoso AF Ferreira A Costa J Costa D Fernandes D . What recovery position should patients adopt after percutaneous liver biopsy? Eur J Gastroenterol Hepatol. (2019) 31:253–9. 10.1097/MEG.0000000000001290 30358572

[11] Hyun CB Beutel VJ. Prospective randomized trial of post-liver biopsy recovery positions: does positioning really matter? J Clin Gastroenterol. (2005) 39:328–32. 10.1097/01.mcg.0000155139.58186.35 15758628

[12] Knauer CM. Percutaneous biopsy of the liver as a procedure for outpatients. Gastroenterology. (1978) 74:101–2. 10.1016/0016-5085(78)90363-3 618416

[13] Neuberger J Patel J Caldwell H Davies S Hebditch V Hollywood C . Guidelines on the use of liver biopsy in clinical practice from the British Society of Gastroenterology, the Royal College of Radiologists and the Royal College of Pathology. Gut. (2020) 69:1382–403. 10.1136/gutjnl-2020-321299 32467090 PMC7398479

[14] Castéra L Nègre I Samii K Buffet C. Patient-administered nitrous oxide/oxygen inhalation provides safe and effective analgesia for percutaneous liver biopsy: a randomized placebo-controlled trial. Am J Gastroenterol. (2001) 96:1553–7. 10.1111/j.1572-0241.2001.03776.x 11374698

[15] Grant A Neuberger J. Guidelines on the use of liver biopsy in clinical practice. British Society Gastroenterol Gut. (1999) 45:Iv1–iv11. 10.1136/gut.45.2008.iv1 10485854 PMC1766696

[16] Eisenberg E Konopniki M Veitsman E Kramskay R Gaitini D Baruch Y. Prevalence and characteristics of pain induced by percutaneous liver biopsy. Anesth Analg. (2003) 96:1392–6. Epub 2003/04/23. PubMed PMID: 12707140. 10.1213/01.ANE.0000060453.74744.17 12707140

[17] Filingeri V Sforza D Tisone G. Complications and risk factors of a large series of percutaneous liver biopsies in patients with liver transplantation or liver disease. Eur Rev Med Pharmacol Sci. (2015) 19:1621–9. 26004602

[18] Froehlich F Lamy O Fried M Gonvers JJ. Practice and complications of liver biopsy. Results of a nationwide survey in Switzerland. Dig Dis Sci. (1993) 38:1480–4. 10.1007/BF01308607 8344104

[19] Caldwell SH. Controlling pain in liver biopsy, or “we will probably need to repeat the biopsy in a year or two to assess the response”. Am J Gastroenterol. (2001) 96:1327–9. 10.1016/S0002-9270(01)02410-8 11374664

[20] Tobin MV Gilmore IT. Plugged liver biopsy in patients with impaired coagulation. Dig Dis Sci. (1989) 34:13–5. 10.1007/BF01536147 2910671

[21] Bala MM Riemsma RP Wolff R Kleijnen J. Microwave coagulation for liver metastases. Cochrane Database Syst Rev. (2013) 10:Cd010163. 10.1002/14651858.CD010163.pub2 24122576

[22] Lindor KD Bru C Jorgensen RA Rakela J Bordas JM Gross JB . The role of ultrasonography and automatic-needle biopsy in outpatient percutaneous liver biopsy. Hepatology. (1996) 23:1079–83. 10.1002/hep.510230522 8621137

[23] Tan KT Rajan DK Kachura JR Hayeems E Simons ME Ho CS. Pain after percutaneous liver biopsy for diffuse hepatic disease: a randomized trial comparing subcostal and intercostal approaches. J Vasc Interv Radiol. (2005) 16:1215–9. 10.1097/01.RVI.0000173282.14018.79 16151062

[24] Fernández-Salazar L Velayos B Aller R Lozano F Garrote JA González JM. Percutaneous liver biopsy: patients' point of view. Scand J Gastroenterol. (2011) 46:727–31. 10.3109/00365521.2011.558112 21366386

[25] Cadranel JF Rufat P Degos F. Practices of liver biopsy in France: results of a prospective nationwide survey. For the Group of Epidemiology of the French Association for the Study of the Liver (AFEF). Hepatology. (2000) 32:477–81. 10.1053/jhep.2000.16602 10960438

[26] Minuk GY Sutherland LR Wiseman DA MacDonald FR Ding DL. Prospective study of the incidence of ultrasound-detected intrahepatic and subcapsular hematomas in patients randomized to 6 or 24 hours of bed rest after percutaneous liver biopsy. Gastroenterology. (1987) 92:290–3. 10.1016/0016-5085(87)90119-3 3539691

